# Evaluation of Isolation Area, Myocardial Injury and Left Atrial Function Following High-Power Short-Duration Radiofrequency or Second-Generation Cryoballoon Ablation for Atrial Fibrillation

**DOI:** 10.3390/jcdd9100327

**Published:** 2022-09-28

**Authors:** Krisztian Istvan Kassa, Zsofia Nagy, Daniel Simkovits, Zsuzsanna Kis, Tamas Ferenci, Zoltan Som, Csaba Foldesi, Attila Kardos

**Affiliations:** 1 Karoly Racz Doctoral School of Clinical Medicine, Semmelweis University, 1085 Budapest, Hungary; 2 Gottsegen National Cardiovascular Center, 1096 Budapest, Hungary; 3 Physiological Controls Research Center, Obuda University, 1034 Budapest, Hungary; 4 Department of Statistics, Corvinus University of Budapest, 1093 Budapest, Hungary

**Keywords:** atrial fibrillation, pulmonary vein isolation, high-power short-duration radiofrequency, second-generation cryoballoon, isolation area, left atrial function, myocardial injury

## Abstract

This randomized study aims to compare the left atrial (LA) lesion size, function, and tissue damage following pulmonary vein isolation (PVI) by high-power short-duration (HPSD) radiofrequency (RF) and second-generation cryoballoon (CB2) ablation. We enrolled 40 patients with paroxysmal atrial fibrillation who underwent PVI by HPSD RF (*n* = 21) or CB2 (*n* = 19). Every patient underwent LA CT angiography and transthoracic echocardiography (TTE) to assess the LA anatomy and function. Biomarker levels (hs-cTnT, hs-CRP, LDH) were compared pre- and post-procedurally. Pre- and post-ablation high-density mapping (HDM) was performed. The isolation area was defined under 0.2 mV bipolar voltage (low voltage area, LVA). We calculated the post-PVI LVA/LA surface ratio using LA CT-HDM merge images. At 3-month follow-up, TTE was performed to assess the changes in LA function. Post-ablation hs-cTnT level was significantly higher in the RF group (RF: 1249 ± 469 ng/L, CB2: 995 ± 280 ng/L, *p* = 0.024). Post-PVI hs-CRP (RF: 9.53 ± 10.30 mg/L, CB2: 12.36 ± 5.76 mg/L, *p* = 0.034) and LDH levels (RF: 349.9 ± 65.6 U/L, CB2: 451.6 ± 91.3 U/L, *p* < 0.001) were significantly higher following CB2 ablation. Post-PVI LVA/LA surface ratios were 8.37 ± 6.42% in the RF group and 13.58 ± 8.92% in the CB2 group (*p* = 0.022). LA function did not change significantly after the PVI procedure. Our data indicate that second-generation cryoballoon ablation produces a significantly larger LA lesion size compared to “point-by-point” HPSD radiofrequency. Both techniques preserve LA function. The myocardial component of tissue loss appears to be higher using HPSD radiofrequency ablation, with less collateral damage.

## 1. Introduction

Pulmonary vein isolation (PVI) is the primary endpoint of atrial fibrillation (AF) catheter ablation (CA) procedures [1]. Contact force-sensing radiofrequency (RF) and second-generation cryoballoon (CB2) ablation have comparable efficacy and safety profiles [2], but differ in the energy source and application modes. The “point-by-point” contact force-sensing RF ablation using an electroanatomic mapping system enables the operator to apply lesion sets that fit the pulmonary vein (PV) anatomy and provides information about the quality and location of ablation lesions. The “CLOSE” protocol provides region-specific criteria of lesion contiguity and lesion depth for contact force-sensing RF ablation [3]. The recently used high-power short-duration (HPSD) technique seems to provide more predictable lesion formation with a non-inferior safety profile. HPSD ablation lesions may be broader and slightly shallower, potentially leading to less collateral damage than the standard ablation lesions [4]. Nevertheless, CB2 allows for a simplified and less time-consuming procedure using pulmonary venography to define the tissue contact around the PV ostium. The balloon’s spherical nature and relatively fixed shape produce less accurately applied ablation lesions around the PVs, potentially resulting in more extensive lesion formation. There is no satisfying evidence regarding LA tissue impairment after HPSD radiofrequency and CB2 ablation. This study aimed to compare the LA lesion size, biomarkers of tissue damage, and left atrial mechanical function following the CA of AF using HPSD RF or the CB2 technique.

## 2. Materials and Methods

### 2.1. Study Design and Patient Selection

This is a prospective, randomized, single-center study. A total of 40 patients were enrolled with drug-refractory, symptomatic paroxysmal AF for index PVI achieved by HPSD RF or CB2. The research was conducted between November 2018 and February 2020 at the Gottsegen National Cardiovascular Center. Echocardiographic measurement of the LA parameters and function, serum biomarker assessment, and high-density electroanatomic mapping were performed before and after each PVI procedure. During a 18-month follow-up period, the LA function and arrhythmia-free survival were observed. This study complies with the Declaration of Helsinki and was approved by the National Ethics Committee. Written informed consent was obtained from all patients.

### 2.2. Pre-Procedural Workflow

All patients received direct oral anticoagulant (DOAC) therapy for 4 weeks before the procedure. Anticoagulation was interrupted the day before the PVI procedure. Antiarrhythmic drug (AAD) administration was not altered before ablation.

Computed tomography angiography (CTA) using a 256-slice CT scanner (GE Revolution, GE Healthcare, Chicago, IL, USA) of the LA was performed in all patients to preview the anatomy of the LA and PVs. The CTA images were imported into the Cartoseg CT module (Biosense Webster, Diamond Bar, CA, USA). Segmentation of the LA and PVs was performed, and the 3D reconstruction images were then exported into the real-time mapping system (CARTO3, Biosense Webster, Diamond Bar, CA, USA).

Transthoracic echocardiography (TTE) was performed with a commercially available system (EPIQ 5, Philips, Andover, MA, USA) to measure the LA parameters [LA length, end-diastolic area (EDA), end-systolic area (ESA), end-diastolic volume (EDV), end-systolic volume (ESV), fractional area change (FAC)], and left ventricular (LV) systolic and diastolic function [left ventricular ejection fraction (LVEF, mitral E, A, and E/e’)]. The LV end-diastolic volume (LV EDV), LV end-systolic volume (LV ESV), LV ejection fraction (LV EF), and LA volumes were determined using the biplane Simpson′s method. The left atrial fractional area change (LA FAC) was calculated by the difference in the LA end-diastolic area (LA EDA) and LA end-systolic area (LA ESA) in the apical 4-chamber plane.

All patients underwent transesophageal echocardiography 24 h before the ablation to rule out the presence of thrombi in the LA and left atrial appendage (LAA). Blood samples were taken just before the procedure. The following serum biomarkers were quantified: high-sensitive cardiac troponin-T (hs-cTnT), high-sensitive C-reactive protein (hs-CRP), and lactate dehydrogenase (LDH). Pre-procedural biomarker levels were in the normal range in all patients.

### 2.3. Procedural Workflow

Patients were randomly assigned to either RF or CB2 ablation. All procedures were performed under deep sedation utilizing fractionated boluses of fentanyl and midazolam with the preservation of spontaneous breathing and continuous monitoring of oxygen saturation.

During the radiofrequency ablation procedure, after the first fluoro and/or intracardiac echocardiography (ICE) guided transseptal puncture with the Brockenbrough needle, SL0 sheath (St. Jude Medical, St Paul, MN, USA) was located in the LA and a multipolar, steerable mapping catheter (PentaRay, Biosense Webster, Diamond Bar, CA, USA) was inserted. Before the isolation of the PVs, HDM was completed using the CARTO3 mapping system to rule out any pre-procedural LA low voltage area (LVA). Then, from another femoral venous puncture, the steerable 8.5 Fr long sheath (Agilis TM NxT, St. Jude Medical, St Paul, MN, USA) was passed over into the LA with the “sliding technique” [5] to introduce the contact force-sensing ablation catheter (Navistar Thermocool SmartTouch, Biosense Webster, Diamond Bar, CA, USA) into the LA. The LA–PV junction was identified by anatomy (based on the LA CTA images), impedance, and local electrogram characteristics. RF applications were created around the LA–PV junction according to the CLOSE protocol [3]. A power-controlled mode with 50 W was used. The target ablation index (AI) was 400 on the posterior wall and 550 on the anterior regions of the LA with a 15–20 g contact force and 3 mm lesion-tag size.

Institutional PVI workflow using the CB2 has been previously described [6]. In brief, CB2 ablation was performed with a 28 mm, second-generation single-shot device (Arctic Front Advance, Medtronic, Minneapolis, MN, USA). After the LA mapping procedure, the SL0 sheet was changed over the wire to a 12 Fr steerable sheath (FlexCath Advance, Medtronic, Minneapolis, MN, USA), and the cryoballoon was introduced into the LA. A circular mapping catheter (Achieve Mapping Catheter, Medtronic, Minneapolis, MN, USA) with the CB2 was positioned in each PV ostium. Contrast injection was applied to verify occlusion. A strategy of 180–240 s of freezing was used with a minimum temperature of −60 °C.

PV isolation was guided by a circular mapping catheter (LassoNav, Biosense Webster Inc., Diamond Bar, CA, USA, and Achieve, Medtronic, Minneapolis, MN, USA) in both groups. When the entrance block was confirmed, pacing from all of the bipoles on the circular mapping catheter was performed to assess the exit block by using a pacing stimulus of 10 mA at 2 ms. PV capture without conduction to the LA was considered as proof for an exit block. The procedures were stopped immediately after initial isolation; adenosine was not administered.

During each procedure, CTA reconstruction images of the LA were registered using the mapping system’s image processing module. First, landmark registration was performed with 3–4 landmarks being placed along the PV ostia. Additionally, surface registration was applied to increase the registration accuracy. Following the ablation, high-density voltage remapping was performed using multipolar catheters in all patients. The acquired maps were later used to evaluate the isolation areas.

### 2.4. Post-Procedural Workflow

Following an average of 19.49 ± 2.49 h after each procedure, blood samples were taken and serum markers of myocardial injury and inflammation were quantified. Post-procedural anticoagulation was started 4–6 h after the sheath removal with DOAC. Anticoagulation therapy was continued after the procedure for 3 months, and then according to the CHA_2_DS_2_-VASc score of the patient. AADs were continued for 3 months after the CA, and were then stopped.

### 2.5. Evaluation of Lesion Size

**Figure 1:**
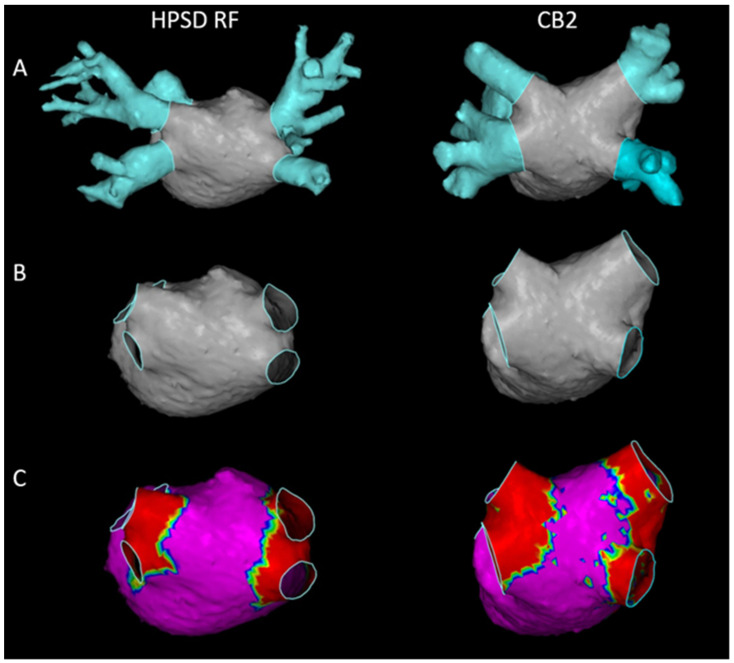
The left atrial (LA) surface and low voltage area (LVA) on the post-PVI high-density mapping (HDM)-computed tomography angiography (CTA) merge images. **Left** side: High-power short-duration radiofrequency (HPSD RF) group. **Right** side: Second-generation cryoballoon (CB2) group. (**A**) CTA image of the LA with the pulmonary veins (PVs), the left atrial appendage (LAA) and the mitral ostium. (**B**) “Clean” LA surface following the extraction of the PVs, the LAA, and the mitral ostium. (**C**) Isolation area represented by LVA following CLOSE-protocol guided “point-by point” HPSD RF (**left**) and CB2 (**right**) ablation. The clean LA surface and the LVA were quantified with an “area measurement” application.

Blinded to the PVI strategy, previously acquired and segmented CTA scan images were imported into the CartoMerge image processing software. Following the extraction of the PVs, the mitral ostium and the LAA, the clean LA surface was quantified with an “area measurement” application. Next, landmark and surface registration was applied using the post-PVI endocardial bipolar voltage remaps. Match statistics of the average distance between the registered CTA and HDM surfaces were recorded. LVA was defined under 0.2 mV bipolar voltage [7]. Thereafter, LVA was measured on the merged post-PVI HDM-CTA scan surface (Figure 1).

### 2.6. Follow-Up

**Figure 2:**
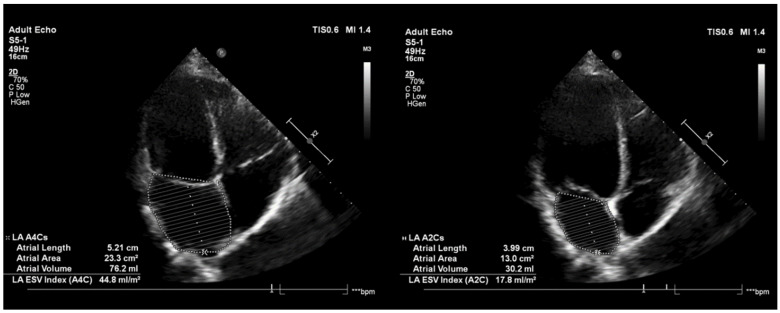
The evaluation of post-procedural LA function by echocardiography.

All patients were recalled for a clinical visit at 3, 6, 12, and 18 months following the PVI procedure. A 12-lead ECG and 24-h Holter ECG were performed on each occasion. At the 3-month follow-up visit, echocardiography was performed to assess changes in the LA parameters and function (Figure 2). Non-scheduled visits were performed in the case of AF suggestive symptoms. Episodes of atrial arrhythmias longer than 30 s were considered as an arrhythmia recurrence.

### 2.7. Statistical Analysis

Continuous variables are presented as the mean ± standard deviation and were compared among groups with the Wilcoxon–Kruskal–Wallis test, categorical variables were presented as frequency (percentage) and were compared among groups using the chi-square test. Differences in the follow-up values of the parameters were investigated using a multivariate regression model, with the post-value being the outcome, and the pre-value (baseline) was used as a covariate in addition to the type of ablation, and further controlling for age (i.e., an ANCOVA-model was used). Continuous variables—that is, the baseline value and age—were expanded with splines to allow for a potentially non-linear effect. The LVA/LA surface ratio was investigated with beta regression and with a generalized linear model using quasibinomial response distribution with log link function (using the same covariates).

## 3. Results

### 3.1. Pre-Procedural Findings

From November 2018 to February 2020, a total of 40 paroxysmal AF patients [16 (40%) women, mean age = 55.9 ± 12.4 years] were included in our study. Every patient was in sinus rhythm before the procedure. Pre-procedural LA scar tissue was not present in any of the patients. Every patient completed the whole follow-up period. Baseline demographic data are shown in Table 1.

### 3.2. Procedural Results

Complete PVI was achieved in all patients (HPSD RF: 21 patients, CB2: 19 patients). The procedure time (included pre-and post-procedural mapping time) was similar between the two groups (HPSD RF: 107.2 ± 30.0 min, CB2: 95.7 ± 20.3 min, *p* = 0.303). The ablation time was significantly higher in the HPSD RF group (ablation time, HPSD RF: 27.93 ± 9.5 min, CB2: 20.16 ± 6.72 min, *p* = 0.001). Fluoroscopy time and radiation exposure were significantly higher in the CB2 group (fluoroscopy time, HPSD RF: 5.62 ± 4.31 min, CB2: 13.65 ± 5.18 min, *p* < 0.001, radiation exposure, HPSD RF: 232 ± 406 cGycm^2^, CB2: 1819 ± 1669 cGycm^2^, *p* < 0.001, Table 2). The mean number of radiofrequency applications was 32 ± 6.0 on the left PVs and 40 ± 8.0 on the right PVs. In the CB2 group 6.26 ± 2.86 freeze circles were used per patient. A small pericardial effusion was found in one case in the HPSD RF group. Transient phrenic nerve paresis occurred once in the CB2 group. None of these complications required further intervention.

### 3.3. Post-Procedural Biomarker Findings

The post-PVI hs-cTnT level was significantly higher in the HPSD RF group (HPSD RF: 1249 ± 469 ng/L, CB2: 995 ± 280 ng/L, *p* = 0.024). The serum Hs-CRP (HPSD RF: 9.53 ± 10.30 mg/L, CB2: 12.36 ± 5.76 mg/L, *p* = 0.034) and LDH levels (HPSD RF: 349.9 ± 65.6 U/L, CB2: 451.6 ± 91.3 U/L, *p* < 0.001) were significantly higher following CB2 ablation (Table 3).

### 3.4. Post-Procedural LA Function by Echocardiography

Left ventricular ejection fraction (LV EF) was preserved in the HPSD RF as well as in the CB2 group. At the 3-month follow-up, the LA FAC was 33.5 ± 10.8% and 35.3 ± 12.3% in the two groups, respectively. Changes in LA FAC were non-significant (*p* = 0.808 and 0.319) in both groups. The baseline LA diameter measured in the parasternal long-axis plane (PLAX) was 56.8 ± 6.6 mm and 58.1 ± 5.2 mm in the HPSD RF and CB2 groups. At the 3-month follow-up, the LA diameter was 53.9 ± 5.0 mm in the HPSD RF group vs. 55.8 ± 5.3 mm in the CB2 group. At baseline, in the HPSD RF group, the LA EDV and ESV were 77.3 ± 34.8 mL and 35.8 ± 13.5 mL, respectively. At the 3-month follow-up, LA EDV in the HPSD RF group was 67.6 ± 18.4 mL, and LA ESV was 34.9 ± 14.8 mL. However, these changes were non-significant (*p* = 0.196 and *p* = 0.102), but there was a slight tendency toward a decrease in LA volumes. Absolute change in thLA EDV, LA ESV, and LA EF at 3-months was also not significant between the two groups (*p* = 0.408, *p* = 0.129, *p* = 0.671, respectively). The echocardiographic results are shown in Table 4.

### 3.5. Comparison of Isolation Areas

**Figure 3:**
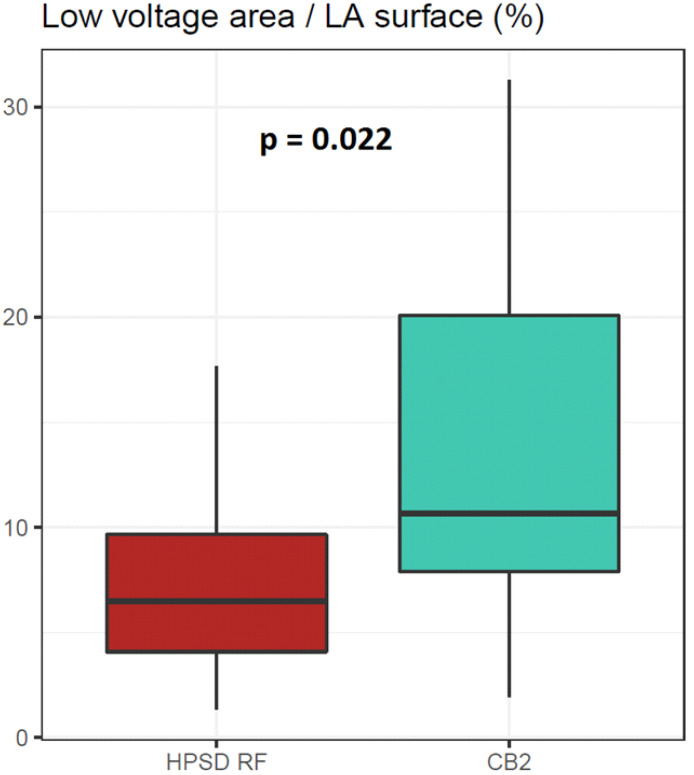
The difference between the LVA/LA surface ratios following HPSD RF and CB2 ablation (*p* = 0.022).

There was no significant difference in the quality of image integration, (match statistics, HPSD RF: 3.559 ± 0.951 mm, CB2: 3.706 ± 1.063 mm, *p* = 0.74). The clean LA surfaces (excluded PVs, mitral ostium, LAA) were comparable following the area measurements based on the CTA images (HPSD RF: 100.1 ± 18.5 cm^2^, CB2: 95.9 ± 18.1 cm^2^, *p* = 0.472). LVA on the post-HPSD RF maps was 8.45 ± 6.60 cm^2^, and it was 12.80 ± 8.02 cm^2^ on the post-CB2 maps (*p* = 0.039). The LVA/LA surface ratio was significantly higher following CB2 ablation (HPSD RF: 8.37 ± 6.42, CB2: 13.58 ± 8.92, *p* = 0.022, Figure 3, Table 5).

### 3.6. Freedom from Arrhythmia

After an 18-month follow-up period, the clinical success rate of the PVI procedures was similar in the two ablation groups (HPSD RF: 81.0%, CB2: 84.2%, *p* = 0.787). Note, however, that this comparison had very low power due to the small number of events.

## 4. Discussion

To the best of the authors’ knowledge, this is the first study that compared the lesion size, myocardial damage, and post-procedural LA function following PVI by HPSD RF using the CLOSE protocol and CB2 ablation.

The main findings of this study are as follows. In patients with paroxysmal AF, (1) the CLOSE-protocol guided HPSD RF ablation produces smaller isolation area than the 28-mm CB2; (2) both HPSD RF and CB2 ablation techniques preserve LA function; and (3) the statistically significant differences in biomarker levels support that the myocardial component of tissue loss may be higher using HPSD RF ablation with less inflammation and collateral injury compared to CB2.

### 4.1. Procedural Data

Procedure time and radiofrequency time using the HPSD RF ablation were similar to previous reports on the HPSD settings [4]. The procedure time was comparable between the HPSD RF and CB2 groups. However, high-density mapping was performed before and after every cryoablation, which lengthened the procedural duration. When analyzing the cryoablation procedures, we observed a mean number of 6.26 ± 2.86 applications for all PVs, and 1.57 freezing applications per vein ratio. Comparing the HPSD RF and CB2 groups, both the fluoroscopy time and radiation exposure were higher in the CB2 group.

### 4.2. Serum Biomarkers as Indicators of LA Damage

Numerous studies have published various results on serum biomarkers as indicators of myocardial injury following PVI using different ablation techniques. Kühne et al. reported a higher rise in the TnT levels with radiofrequency ablation using a maximum of 35 W energy compared to cryoballoon ablation with two, 5-min freezing circles. They concluded that a higher rise in TnT with RF ablation indicates more myocardial injury compared to cryoballoon ablation [8]. Siklódy et al. found comparable TnT and CRP levels using similar ablation settings [9]. They suggested that the lesion size and not the ablation method appeared to be responsible for the magnitude of inflammation. Casella et al. observed greater TnI levels following CB2 ablation compared to other techniques. They found that there was a significant correlation between the TnI levels and longer energy delivery, and elevated levels of cardiac biomarkers did not translate into a better procedural outcome [10]. Hisazaki et al. compared the serum biomarkers of inflammation, myocardial and endothelial injury after PVI using contact force sensing RF catheters and CB2 devices [11]. The RF current was delivered with a power of up to 35 W, its contact force greater than 10 g, and two 3-min freezes were applied during cryoablation. They reported that cryoablation resulted in a greater amount of myocardial injury with a similar inflammatory response. Their findings are nearly consistent with a prior study presented by Yano et al. [12]. They enrolled 263 paroxysmal AF patients and demonstrated that the serum hs-TnI level was significantly lower and the CRP level was significantly higher after PVI by the low-power long-duration (LPLD) RF strategy (maximum power: 30 W for 25 s) than that of the one by cryoballoon (CB) ablation (freeze time 180–240 s, applications for PVs: 6.0 ± 1.8). Therefore, CB ablation caused more myocardial injury than RF ablation, and at the same time, RF ablation caused more inflammation than CB ablation. However, these phenomena did not affect the recurrence of AF.

In this study, we used contact force-sensing RF catheters and HPSD settings (power-controlled mode with 50 W, posterior wall AI: 400 and other regions AI: 550). The target contact force was 15–20 g and we encircled the ostia of the PVs according to the CLOSE-protocol. Cryoablation was performed by a CB2 with a strategy of 180–240 s of freezing and a 1.57 freeze application per vein ratio. Our study demonstrated that HPSD RF ablation resulted in significantly higher hs-cTnT and lower CRP and LDH levels than the CB2. Although the current study was based on a small sample of participants, the findings suggest that the HPSD technique enables the production of effective, transmural lesions while minimizing inflammation and collateral injury. This finding may be due to the lesion characteristics of HPSD ablation. As multiple ex vivo studies have demonstrated, high-energy ablation for a shorter period results in different lesion geometry (larger maximum diameter, combined with smaller lesion depth) and less collateral damage than LPLD ablation [4,13]. Note, however, that lesion formation and the mechanisms of tissue damage markedly differed in the RF and cryoablation regardless of the RF power settings.

### 4.3. LA Function Defined by Echocardiography Measurements

Changes in LA-EF were non-significant either in the HPSD RF and in the CB2 group, or between the two groups. According to the LV filling parameters, diastolic LV function was not heavily deteriorated. HPSD RF and CB2 ablation is associated with scar tissue formation, which, based on the literature data, may alter the LA function. In certain cases, proven by MR examination, a decrease in the LA function can be found [14]. In our study of patients, LA EF did not deteriorate in any of the two groups. Absolute change in LA function and LA dimensions were non-significant in both groups, which may be due to the patient characteristics—paroxysmal AF, preserved LVEF, at the most mild atrial dilatation and mitral regurgitation—and size of the study population. Our findings correlate to a recent study by Giannopoulos et al. [15], who defined post-ablation LA function following PVI achieved by CRF or CB2 using 3D echocardiography.

### 4.4. Isolation Area after HPSD RF and CB2 Ablation

Endocardial bipolar voltage mapping is an alternative way of LA scar assessment with a good correlation between voltage maps and MRI scar maps [16]. Its performance can be improved by an automated collection of a large number of points and using mapping catheters with dense, small electrodes [17]. We selected a cut-off value of 0.2 mV for the definition of LVA. Although a less “strict” 0.5 mV value is also commonly used, we wanted to ensure a clear boundary between the ablated and intact tissue. Numerous studies have attempted to evaluate isolation areas following PVI using electroanatomic mapping. Kenigsberg et al. observed wide and antral lesion formation following PVI procedures by 28-mm CB2 [18]. They assessed 43 AF patients right after cryoablation and found that 27% of the LA posterior wall remained unablated. Takahashi et al. reported larger low-voltage zones following CB2 ablation than the one with contact force-sensing RF [19]. They enrolled 40 patients also with persistent and paroxysmal AF. Even though this was the first actual comparison of PVI techniques regarding lesion size, they applied touch-up RF ablations during cryoablation and used different mapping systems for the assessment of LVA. In another major study, Chikata et al. found that contact force-sensing RF ablation resulted in a larger isolation area than CB2 ablation [20]. They used HDM with multielectrode diagnostic catheters and merged LA CTA images using the CARTO3 system. They demonstrated that the RF ablation zone correlated positively with the LA surface area. As the size of the balloon was fixed, the lesions generated around the PV antra did not widen, regardless of the increment in the LA size. Unlike in the RF group, the operator could freely design the ablation line. In contrast, our RF PVI protocol was based on anatomical characteristics, striving to ablate on the LA–PV junction.

### 4.5. Clinical Implications

In our study, the isolation area by CB2 was greater than the one by HPSD RF, while the myocardial damage was larger in the HPSD group. We found that the overall LA tissue damage was not extensive by either HPSD RF or CB2 ablation and that the post-ablation LA function and the 18-month success rates were similar [21,22]. Previous studies have described that extended iatrogenic left atrial fibrosis correlates with increased LA pressure, AF recurrence, and stiff LA syndrome [23,24]. The results of our study are reassuring given the fact that PVI is emerging as first line therapy for AF. Moreover, it has been shown that the procedure performed at an early stage might delay the progression of AF [25].

### 4.6. Limitations

The main limitations of our study are the small sample size and the fact that the high-density voltage remapping was performed right after the procedures. The isolation circle around the PV ostia might vary, depending on the preferences of the operator. Additional LA function parameters such as the strain and strain rates were not assessed. Our findings should be confirmed with a larger patient population.

## 5. Conclusions

This randomized study directly assessed and compared the impact of the HPSD radiofrequency and 28-mm CB2 ablation of paroxysmal AF. In conclusion, the extent of the post-PVI lesion size was significantly higher following CB2 ablation compared to “point-by-point” HPSD RF ablation. Both the HPSD RF and CB2 techniques preserved LA function. The myocardial component of tissue loss appeared to be higher using HPSD RF ablation. The 18-month clinical success rate was similar.

## Figures and Tables

**Table 1:** The baseline demography.

Baseline Demography and Pre-Procedural Findings				
		HPSD RF (*n* = 21)	CB2 (*n* = 19)	*p* Value
Women, *n* (%)		10 (48)	6 (32)	0.301
Age (years)		59.5 ± 12.3	51.9 ± 11.5	0.071
Duration of AF (months)		57.6 ± 66.9	77.1 ± 63.5	0.102
Antiarrhythmic drugs (AAD), *n* (%)	sotalol	4 (19)	2 (11)	0.530
	amiodarone	2 (10)	3 (16)	
	propafenon	3 (14)	8 (42)	
	beta-blocker	16 (76)	11 (58)	
	none	1 (5)	2 (11)	
Sinus rhythm prior ablation, *n* (%)		21 (100)	19 (100)	1.000
Body mass index (BMI)		27.81 ± 5.69	28.88 ± 5.08	0.620
Hypertension (HT), *n* (%)		9 (45)	10 (53)	0.634
Diabetes mellitus (DM), *n* (%)		3 (15)	1 (5)	0.316
Hyperlipidemia (HLP), *n* (%)		3 (15)	4 (21)	0.622
Thyroid dysfunction *n* (%)		2 (10)	0 (0)	0.157
Glomerular filtration rate (GFR) < 60, *n* (%)		2 (10)	0 (0)	0.367
Left ventricular (LV) ejection fraction (EF) (%)		60.7 ± 6.55	62.11 ± 4.11	0.430
Coronary artery disease (CAD), *n* (%)		2 (10)	1 (5)	0.579
Congestive heart failure (CHF), *n* (%)		1 (5)	0 (0)	0.323
CHA_2_DS_2_-VASc score, *n* (%)	0	5 (25)	7 (37)	0.476
	1	4 (20)	6 (32)	
	2	5 (25)	3 (16)	
	3	3 (15)	0 (0)	
	4	1 (5)	1 (5)	
	5	2 (10)	1 (5)	
	6	0 (0)	1 (5)	

**Table 2:** The procedural results.

Procedural Results			
	HPSD RF (*n* = 21)	CB2 (*n* = 19)	*p* Value
Procedure time (min)	107.2 ± 30.0	95.7 ± 20.3	0.303
Ablation time (min)	27.93 ± 9.5	20.16 ± 6.72	0.001
Fluoroscopy time (min)	5.62 ± 4.31	13.65 ± 5.18	<0.001
Fluoroscopy dose (cGycm^2^)	232 ± 406	1819 ± 1669	<0.001
Number of applications	73 ± 8.0	6.26 ± 2.86	-

The procedure time was comparable (*p* = 0.303) and the ablation time was longer in the HPSD RF group (*p* = 0.001). Fluoroscopy time and fluoroscopy dose were significantly higher in the CB2 group (*p* < 0.001 and *p* < 0.001).

**Table 3:** The post-procedural serum biomarker findings.

Post-Procedural Biomarker Findings			
	HPSD RF (*n* = 21)	CB2 (*n* = 19)	*p* Value
hs-cTnT (ng/L)	1249 ± 469	995 ± 280	0.024
hs-CRP (mg/L)	9.53 ± 10.30	12.36 ± 5.76	0.034
LDH (U/L)	349.9 ± 65.6	451.6 ± 91.3	<0.001

Hs-cTnT: high-sensitive cardiac troponin-T, hs-CRP: high-sensitive C-reactive protein, LDH: lactate dehydrogenase.

**Table 4:** The post-procedural LA function by echocardiography.

Post-Procedural LA Function by Echocardiography							
LA Parameters	HPSD RF (*n* = 21)			CB2 (*n* = 19)			Difference at 3-Month FU
	Pre-PVI	3-Month FU	*p* Value	Pre-PVI	3-Month FU	*p* Value	*p* Value
LA size long (mm)	56.8 ± 6.6	53.9 ± 5.0	0.325	58.1 ± 5.2	55.8 ± 5.3	0.148	0.572
LA max area (cm^2^)	22.6 ± 4.1	22.2 ± 3.6	0.778	23.4 ± 3.6	24.3 ± 4.1	0.307	0.235
LA min area (cm^2^)	14.9 ± 3.7	14.8 ± 3.6	0.845	14.7 ± 3.8	15.9 ± 4.6	0.211	0.633
FAC	33.7 ± 13.3	33.5 ± 10.8	0.808	38.1 ± 8.7	35.3 ± 12.3	0.525	0.319
Peak E/A	1.7 ± 2.6	1.2 ± 0.4	0.038	1.3 ± 0.4	1.1 ± 0.2	0.855	0.287
LA EDV (ml)	77.3 ± 34.8	67.6 ± 18.4	0.196	69.2 ± 20.1	74.0 ± 19.9	0.164	0.408
LA ESV (ml)	35.8 ± 13.5	34.9 ± 14.8	0102	34.2 ± 13.6	37.5 ± 17.0	0.348	0.129
LA EF (%)	50.2 ± 19.7	49.6 ± 14.8	0.903	49.7 ± 15.5	50.8 ± 13.4	0.847	0.671
E	64.7 ± 15.6	67.1 ± 20.7	0.382	72.8 ± 18.1	68.5 ± 16.7	0.599	0.238
E’	10.4 ± 2.5	10.0 ± 3.5	0.395	12.5 ± 3.5	10.9 ± 3.1	0.105	0.568
E/E’	6.6 ± 2.6	7.32 ± 3.1	0.326	6.2 ± 1.9	6.6 ± 1.9	0.121	0.972

EDV: end-systolic volume, ESV: end-systolic volume, EF: ejection fraction, FU: follow-up. *p*-values for within-group comparison are based on a paired Wilcoxon-test; they are univariate comparisons without controlling for any confounder. The final *p*-value pertains to the difference between groups in the change over time; this was obtained from an ANCOVA-model, controlling for age.

**Table 5:** The LA surface and LVA measurements.

LA Surface and LVA Measurements			
	HPSD RF (*n* = 21)	CB2 (*n* = 19)	*p* Value
Number of points per map	1748 ± 477.7	1660 ± 477.3	0.606
Match statistics (mm)	3.559 ± 0.951	3.706 ± 1.063	0.740
Left atrial (LA) surface (cm^2^)	100.1 ± 18.5	95.9 ± 18.1	0.472
Normal voltage area (NVA) (cm^2^)	92.1 ± 18.1	83.1 ± 19.4	0.170
Low voltage area (LVA) (cm^2^)	8.45 ± 6.60	12.80 ± 8.02	0.039
LVA/LA surface (%)	8.37 ± 6.42	13.58 ± 8.92	0.022

No difference was observed in the quality of electroanatomic mapping and image integration. The clean left atrial (LA) surface was comparable between the two groups.

## Data Availability

The data supporting the findings of this study are available on request from the corresponding author. The data are not publicly available due to privacy or ethical restrictions.
